# Urine proteomic signatures of histological class, activity, chronicity, and treatment response in lupus nephritis

**DOI:** 10.1172/jci.insight.172569

**Published:** 2024-01-23

**Authors:** Andrea Fava, Jill Buyon, Laurence Magder, Jeff Hodgin, Avi Rosenberg, Dawit S. Demeke, Deepak A. Rao, Arnon Arazi, Alessandra Ida Celia, Chaim Putterman, Jennifer H. Anolik, Jennifer Barnas, Maria Dall’Era, David Wofsy, Richard Furie, Diane Kamen, Kenneth Kalunian, Judith A. James, Joel Guthridge, Mohamed G. Atta, Jose Monroy Trujillo, Derek Fine, Robert Clancy, H. Michael Belmont, Peter Izmirly, William Apruzzese, Daniel Goldman, Celine C. Berthier, Paul Hoover, Nir Hacohen, Soumya Raychaudhuri, Anne Davidson, Betty Diamond, the Accelerating Medicines Partnership in RA/SLE network, Michelle Petri

**Affiliations:** 1 Division of Rheumatology, Johns Hopkins University, Baltimore, Maryland, USA.; 2 New York University School of Medicine, New York, New York, USA.; 3 University of Maryland, Baltimore, Maryland, USA.; 4 University of Michigan, Ann Arbor, Michigan, USA.; 5 Division of Renal Pathology, Johns Hopkins University, Baltimore, Maryland, USA.; 6 Division of Rheumatology, Inflammation, and Immunity, Department of Medicine, Brigham and Women’s Hospital, Harvard Medical School, Boston, Maryland, USA.; 7 Feinstein Institutes for Medical Research, Northwell Health, Manhasset, New York, USA.; 8 Università La Sapienza, Rome, Italy.; 9 Albert Einstein College of Medicine, New York, New York, USA.; 10 Azrieli Faculty of Medicine of Bar-Ilan University, Zefat, Israel.; 11 University of Rochester, Rochester, New York, USA.; 12 University of California, San Francisco, San Francisco, California, USA.; 13 Medical University of South Carolina, Charleston, South Carolina, USA.; 14 University of California, San Diego, San Diego, California, USA.; 15 Oklahoma Medical Research Foundation and University of Oklahoma Health Sciences Center, Oklahoma City, Oklahoma, USA.; 16 Division of Nephrology, Johns Hopkins University, Baltimore, Maryland, USA.; 17 Broad Institute, Boston, Maryland, USA.; 18 Centre for Genetics and Genomics Versus Arthritis, Centre for Musculoskeletal Research, Manchester Academic Health Science Centre, The University of Manchester, Manchester, United Kingdom.; 19 The Accelerating Medicines Partnership in RA/SLE network is detailed in Supplemental Acknowledgments.

**Keywords:** Autoimmunity, Nephrology, Diagnostics, Lupus, Proteomics

## Abstract

Lupus nephritis (LN) is a pathologically heterogenous autoimmune disease linked to end-stage kidney disease and mortality. Better therapeutic strategies are needed as only 30%–40% of patients completely respond to treatment. Noninvasive biomarkers of intrarenal inflammation may guide more precise approaches. Because urine collects the byproducts of kidney inflammation, we studied the urine proteomic profiles of 225 patients with LN (573 samples) in the longitudinal Accelerating Medicines Partnership in RA/SLE cohort. Urinary biomarkers of monocyte/neutrophil degranulation (i.e., PR3, S100A8, azurocidin, catalase, cathepsins, MMP8), macrophage activation (i.e., CD163, CD206, galectin-1), wound healing/matrix degradation (i.e., nidogen-1, decorin), and IL-16 characterized the aggressive proliferative LN classes and significantly correlated with histological activity. A decline of these biomarkers after 3 months of treatment predicted the 1-year response more robustly than proteinuria, the standard of care (AUC: CD206 0.91, EGFR 0.9, CD163 0.89, proteinuria 0.8). Candidate biomarkers were validated and provide potentially treatable targets. We propose these biomarkers of intrarenal immunological activity as noninvasive tools to diagnose LN and guide treatment and as surrogate endpoints for clinical trials. These findings provide insights into the processes involved in LN activity. This data set is a public resource to generate and test hypotheses and validate biomarkers.

## Abstract 2

<p>Urine proteomics identifies active intrarenal processes, potential therapeutic targets, and noninvasive biomarkers of lupus nephritis.</p>

## Introduction

Lupus nephritis (LN) is a leading cause of morbidity and mortality in patients with systemic lupus erythematosus (SLE), resulting in end-stage kidney disease in 20% of cases (1), especially in people of non-European descent (2, 3). Despite optimal treatment, only 30%–40% of patients with LN achieve a complete renal response at 1 year (4–6). Thus, there is a pressing need to identify novel treatment strategies to prevent kidney damage and mortality.

LN diagnosis, classification, and treatment rely on kidney biopsies obtained in SLE patients with proteinuria. Kidney biopsies are necessary because proteinuria neither distinguishes treatable inflammation from chronic damage nor differentiates International Society of Nephrology (ISN) LN classes (7). Furthermore, proteinuria does not correlate with intrarenal inflammation and is a lagging indicator as it occurs and persists after damage has ensued. Kidney biopsies also have limitations, including procedure-related complications and sampling error, and may delay diagnosis and treatment. In addition, kidney biopsies are invasive and may be challenging to repeat in all patients with LN. A noninvasive biomarker that reflects intrarenal pathology could lead to early diagnosis and guide treatment by assessing response in real time.

Urine collects the byproducts of kidney pathology. By reflecting intrarenal processes (8, 9), urine proteomics is an ideal noninvasive tool to discover disease mechanisms, identify novel therapeutic targets, and verify noninvasive biomarkers. Previous studies explored urine proteomics in LN but were limited by technical sensitivity, sample size, or cross-sectional design (8, 10–14). Several biomarkers have been identified in LN. However, their performance has been often measured as the ability to discriminate LN from healthy donors or proteinuric LN from LN in remission (15). Such biomarkers provide limited clinical value because they do not outperform readily available biomarkers (i.e., proteinuria) and thus do not impact treatment decisions or prognosis.

Accordingly, this study applied urine proteomics to the large and ancestrally diverse Accelerating Medicines Partnership (AMP) LN longitudinal cohort (16) to define pathways and clinically meaningful biomarkers linked to histology class, LN activity, and response to treatment. We found that markers of neutrophil/monocyte degranulation, macrophage activation, and extracellular matrix remodeling are implicated in proliferative LN (the most aggressive type), LN activity, and response to treatment. Candidate biomarkers were validated and provide potentially treatable targets. This large data set is available to the public for further research.

## Results

### Pipeline and recruitment.

**Figure 1:**
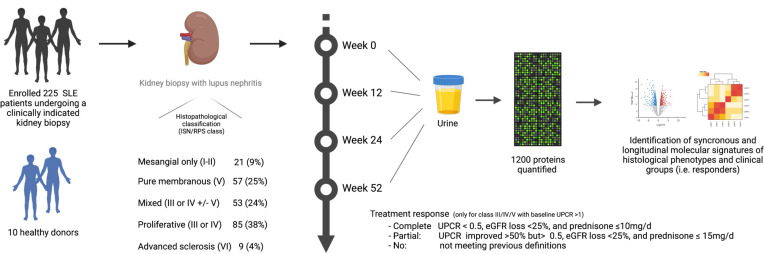
Experimental pipeline. eGFR, estimated glomerular filtration rate.

To characterize LN molecular signatures of specific LN subtypes and treatment response, we analyzed the longitudinal urine proteomic profiles (1,200 proteins) of patients with LN and their clinical and histologic associations (Figure 1).

We recruited 225 patients with SLE who underwent a clinically indicated kidney biopsy and had a urine protein-to-creatinine ratio (UPCR) > 0.5 g/g. To capture LN diversity, we included all patients with LN defined by histology. Most patients (62%) had proliferative LN; 85 (38%) had pure proliferative LN (class III or IV), and 53 (24%) had mixed LN (class III or IV + V). One quarter (25%) had pure membranous LN (class V); finally, 21 (9%) had mesangial limited LN (class I or II), and 9 (4%) had advanced sclerosis (class VI). For comparison, we recruited 10 healthy donors (HDs) without a past medical history of any kidney disease and with negative autoimmune serologies. All patients and controls had a baseline sample. Longitudinal urine samples were collected for patients with class III, IV, or V LN; 136, 109, and 96 samples were analyzed at week 12, 24, and 52, respectively. The baseline demographic and clinical characteristics are summarized by Supplemental Table 1; supplemental material available online with this article; https://doi.org/10.1172/jci.insight.172569DS1 The patients were similar in age and sex. As expected, proliferative LN had higher histological activity (NIH Activity Index). Except for ISN class VI (advanced sclerosis), chronicity was similar in the other classes. Proteinuria at the time of biopsy was lower in class I or II LN (median 0.76 [range 0.5–4]) whereas all other classes were similar, highlighting the inability of proteinuria to distinguish between LN classes, as we have previously shown (7). The EGFR was reduced in all patients with LN compared with HDs, with the lowest values observed in class VI (median 46 mL/min [range 9–63]), followed by proliferative LN (median 88 mL/min [range 12–160]) and pure membranous (median 100 mL/min [range 15–145]). Patients with proliferative or membranous LN were followed longitudinally: complete response rates at week 52 were more common in proliferative LN as compared with pure membranous LN (34% vs. 16%). We assayed a total of 573 urine proteomic profiles from these 225 unique LN patients and 10 HDs.

### Molecular signatures of LN.

**Figure 2:**
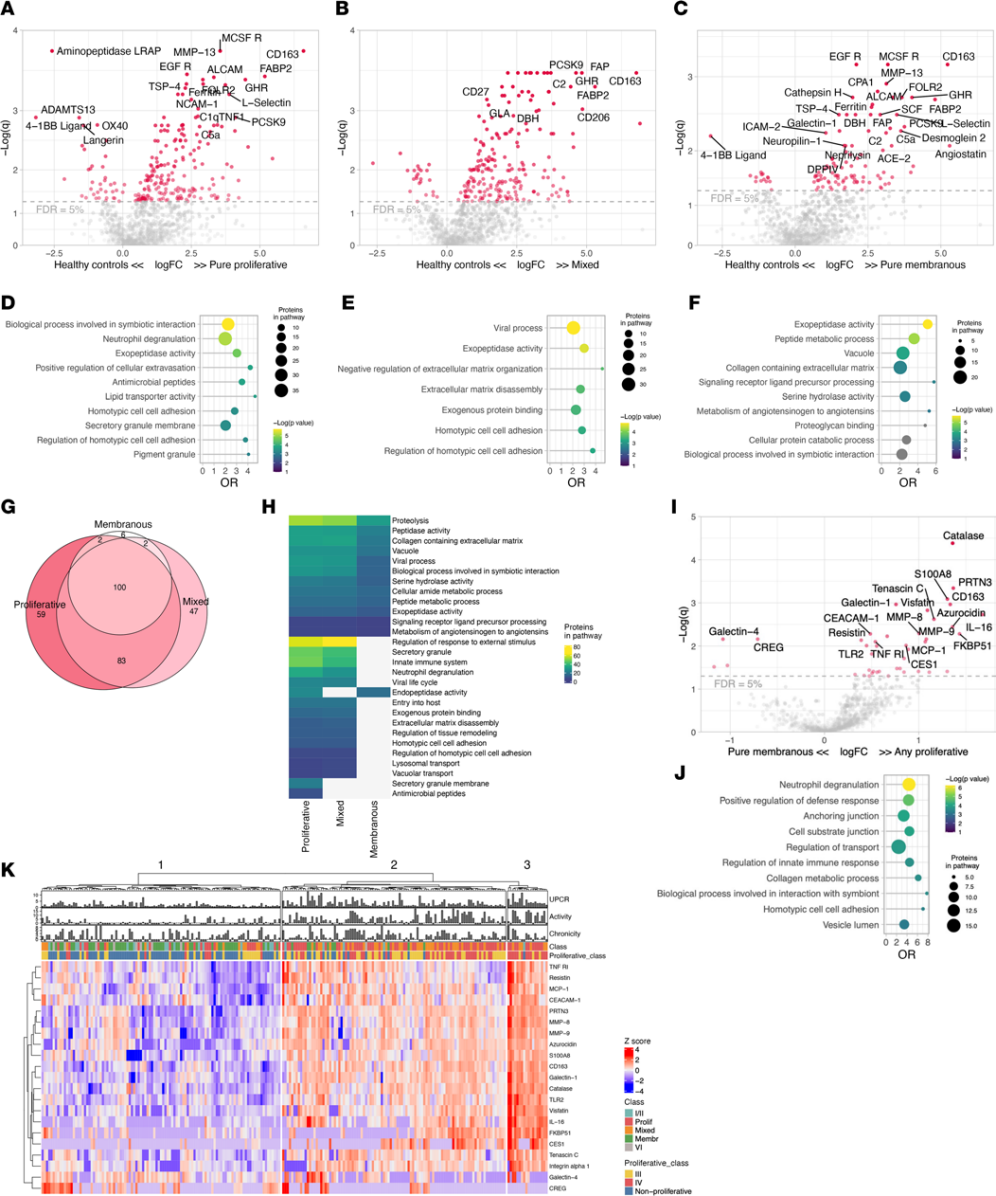
Proteomic signatures of LN histological classes. Volcano plots of the differential urinary protein abundances in pure proliferative (*n* = 85) (**A**), mixed (*n* = 55) (**B**), and membranous (*n* = 55) (**C**) LN compared with healthy controls (*n* = 10). Pathway enrichment analysis of the proteins enriched in pure proliferative (**D**), mixed (**E**), and membranous (**F**) LN (FDR < 5%); pathways in gray had a *q* > 0.05. (**G**) Venn diagram summarizing the shared significantly changed proteins enriched in the 3 classes displayed in **A**–**C**. (**H**) Heatmap summarizing the pathways enriched (FDR < 25%) in the 3 classes. (**I**) Volcano plot displaying the differential urine protein abundances in any proliferative (pure or mixed) and pure membranous with relative pathway enrichment analysis (**J**). (**K**) Heatmap displaying the unsupervised clustering based on the urine abundances of the proteins differentially abundant in any proliferative versus pure membranous (**I**); clinical features are displayed. Not available activity and chronicity scores are indicated as –1. FDR, false discovery rate; OR, odds ratio; *q*, adjusted *P* value (Benjamini-Hochberg).

To identify the proteomic signature of each LN class, we initially compared the urine proteomic profile of LN with HDs without clinical proteinuria (Figure 2, A–C). Hundreds of proteins were significantly increased in all LN classes (Supplemental Data 1). In this study, pure proliferative LN (class III or IV) and mixed LN (class III or IV + V) were often analyzed together because they share the component of “proliferative” LN, which is linked to worse outcomes (17). Accordingly, patients with pure proliferative and mixed LN showed similar proteomic profiles, with pathway enrichment analysis detecting innate immune system activation, neutrophil degranulation, viral life cycle, and extracellular matrix disassembly/protease activity (Figure 2, D–H, and network analysis in Supplemental Figure 1). Most of the proteins enriched in pure membranous LN were also found in proliferative LN, indicating common core pathways across proliferative, mixed, and pure membranous LN (Figure 2G and Supplemental Figure 2). In contrast, most of the proteins enriched in proliferative LN (pure or mixed) were not found in membranous LN, suggesting that distinct biological processes are restricted to proliferative LN. At the pathway enrichment level, all 3 LN groups showed evidence of protease activity and extracellular matrix remodeling (Figure 2H).

Proliferative LN is the most aggressive form of LN and carries a higher risk of permanent kidney damage (17). To better define proliferative LN’s specific pathological pathways, we compared the proteomic profiles of proliferative (pure and mixed) to pure membranous LN. Proliferative LN signature was dominated by higher levels of CD163 (a macrophage marker; fold-change [FC] = 2.5, *q* = 0.001), IL-16 (a proinflammatory chemokine; FC = 3.2, *q* = 0.002), and granulocyte degranulation products such as PR3, S100A8, azurocidin, catalase, and MMP8 (range FC = 2.5–2.6, *q* = 4 × 10^–5^ to 5 × 10^–3^), among many others (Figure 2I and Supplemental Figure 1A). Pathway enrichment analysis verified that neutrophil degranulation was the biological signature most enriched in proliferative LN (Figure 2J). Several macrophage markers, such as CD163, CD206, galectin-1, and FOLR2, were enriched in all classes. The urinary abundance of these proteins was similar in pure and mixed proliferative LN but higher than membranous (Figure 2F and Supplemental Figure 2).

The urine abundance of the proteins differentially expressed in the proliferative LN signature is displayed in the heatmap in Figure 2K. We noted 3 clusters of patients defined by low, medium, or high expression of this signature. As expected, the low (left) cluster included almost exclusively patients with nonproliferative LN. The medium (center) cluster included mostly patients with proliferative LN but also some pure class V, class I/II, and class VI LN. The high (right) cluster identified patients with the greatest expression of the proliferative LN signature and was dominated by patients with proliferative LN. Patients in this cluster had largely class IV and demonstrated the highest activity indices. Of note, there were several patients with histologically nonproliferative LN in the “medium” cluster and 1 in the “high” cluster, indicating heterogeneity in nonproliferative LN. About 18% of pure membranous LN showed strong inflammatory responses with degranulation and monocyte/macrophage activation signatures. These findings indicate a disconnect between the histological classification and the inflammatory activity detected by urine proteomics in several patients.

Together, these findings implicate active neutrophil/monocyte degranulation and macrophage activation in patients with proliferative LN. These signatures identify patients with higher activity indices. Furthermore, protease activity and extracellular matrix degradation characterize both proliferative and pure membranous LN. Importantly, these intrarenal biological processes can be noninvasively quantified in the urine.

### Proteomic signatures of histological activity and chronicity.

**Figure 3:**
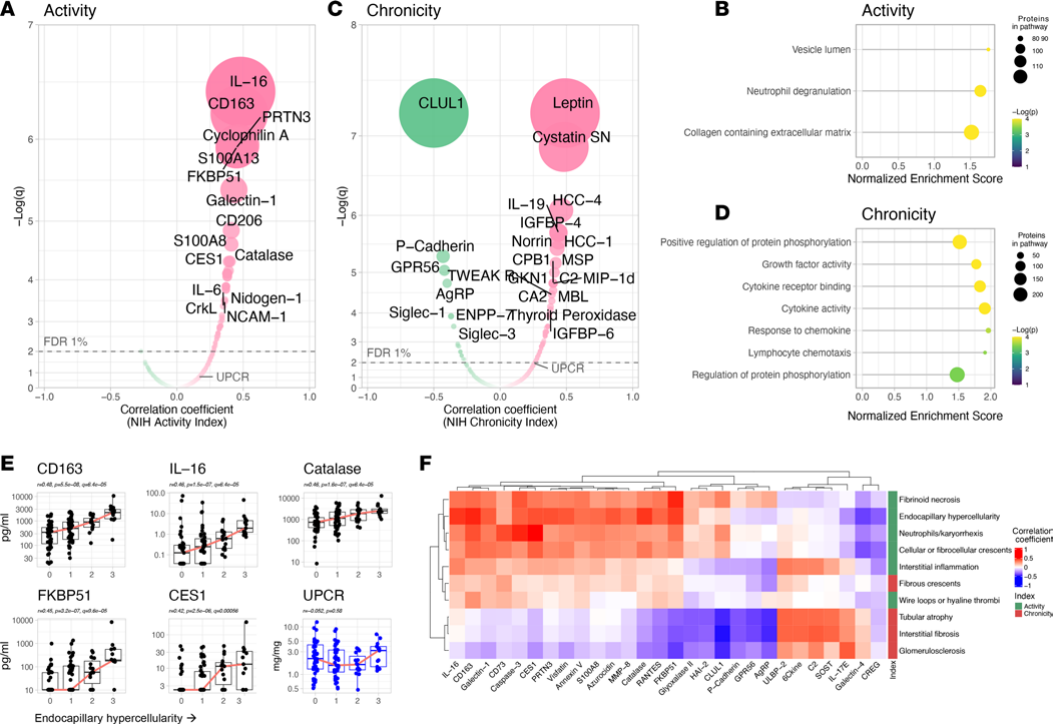
Proteomic signatures of histological activity and chronicity. Volcano plots displaying Pearson’s correlation of the proteins’ urinary abundances and the NIH Activity (**A**) and Chronicity (**C**) indices (*n* = 154). The correlation with the urine protein-to-creatinine ratio (UPCR) is indicated for reference. Pathway enrichment analysis (by gene set enrichment analysis) of the associations of the urinary proteins with the NIH Activity (**B**) and Chronicity (**D**) Indices. (**E**) The 5 most correlated proteins with the endocapillary hypercellularity score are displayed as compared with UPCR. (**F**) Hierarchical clustering based on the correlations of each histological lesion and urinary proteins. All proteins with a strict statistically significant correlation (FDR < 0.01) with at least 1 histological lesion were included. FDR, false discovery rate; *q*, Benjamini-Hochberg adjusted *P* value.

Proliferative LN is heterogeneous in the degree of immunological activity. This is captured by the NIH Activity Index (18). High scores identify more aggressive disease associated with higher risk of kidney failure (17). Five of the 6 components of the NIH Activity Index (endocapillary hypercellularity, neutrophil/karyorrhexis, fibrinoid necrosis, wire loops/hyaline thrombi, and cellular/fibrocellular crescents) are exclusive to proliferative LN (class III or IV ± V), thereby making the NIH Activity Index a quantitative measure of proliferative LN activity. To characterize the pathways and biomarkers of LN activity, we studied the correlation of the urinary proteins with the NIH Activity Index (Figure 3A). We found several urinary proteins directly correlated with the NIH Activity Index, topped by IL-16 and CD163 (Figure 3A). As supported by pathway enrichment analysis (Figure 3B), the signature of LN activity also included proteins associated with degranulation (i.e., PR3, azurocidin, visfatin, MMP8, LAMP1, catalase), macrophage activation (i.e., CD163, CD206, galectin-1), and wound healing/matrix degradation (i.e., nidogen-1, decorin) (Supplemental Figure 3). Importantly, these associations persisted after adjusting for proteinuria, renal fibrosis (NIH Chronicity Index), and treatment in a multivariable model (Supplemental Figure 4). These findings further support the link between proliferative LN and both myeloid cell activity/degranulation and wound healing pathways by demonstrating a direct quantitative association with proliferative LN activity, independent of proteinuria.

Next, we studied the proteomic correlates of intrarenal damage as quantified by the NIH Chronicity Index. The NIH Chronicity Index captures features of irreversible damage, such as interstitial fibrosis and tubular damage, glomerulosclerosis, and fibrous crescents. Figure 3C displays the urinary proteins positively and negatively correlated with intrarenal chronicity. Pathway enrichment analysis identified cytokine/chemokines and growth factor activity (Figure 3D). These associations persisted after adjusting for proteinuria and the NIH Activity Index (Supplemental Figure 4).

### Proteomic signatures of specific histological features.

We analyzed the urinary proteomic profiles of each histological lesion assessed by the NIH Activity and Chronicity Indices (18). In this subanalysis, 115 biopsies with available subscoring were included: 42 (36%) with pure proliferative LN, 33 (29%) with pure membranous LN, and 41 (35%) with mixed LN. The 5 most correlated urinary proteins and each histological feature of the NIH Activity and Chronicity Indices are displayed in Figure 3, E and F, and Supplemental Figure 5. For example, endocapillary hypercellularity, a proliferative LN–defining feature, correlated with urinary CD163, IL-16, catalase, FKBP1, and CES1 (topping several others) but not with proteinuria (Figure 3E). Most lesions shared a similar signature within their respective index (Figure 3F). This is expected since the index components tend to co-correlate. Hierarchical clustering based on urine proteomic signatures revealed that fibrous crescents were more similar to activity-related lesions despite being considered inactive lesions and counted in the NIH Chronicity Index (Figure 3F). Interstitial inflammation (activity), fibrous crescents (chronicity) and, to a lesser extent, wire loops (activity) correlated with biomarkers associated with both active and chronic lesions (Figure 3F). Strikingly, there was no correlation between proteinuria and the histological lesions in the NIH Activity or Chronicity Indices (Figure 3E and Supplemental Figure 5).

### Treatment response is associated with a decline of urinary biomarkers of LN activity, including markers of myeloid immunity and matrix degradation.

Next, we focused on the proteomic signatures linked to treatment response. Complete renal response is currently defined by a decline of UPCR to < 0.5 after 1 or 2 years since it is associated with better long-term preservation of kidney function in LN. To assess response, a baseline UPCR > 1 was required (7). In this analysis, a total of 127 patients were included: 48 (38%) with pure proliferative LN (class III or IV), 41 (32%) with mixed LN (III or IV ± V), and 38 (30%) with pure membranous LN. Response was complete in 34 (27%), partial in 29 (23%), and none in 64 (50%). In this cohort, treatment selection was at the discretion of the treating physician, but mycophenolate mofetil was the mainstay of treatment.

At the time of kidney biopsy (baseline), there was no difference in the urinary proteomic profiles in patients who achieved any clinical response at 1 year (responders) compared to nonresponders (Supplemental Figure 6A), even when the analysis was restricted to patients treated with the same regimen of mycophenolate. Therefore, we focused on longitudinal trajectories.

**Figure 4:**
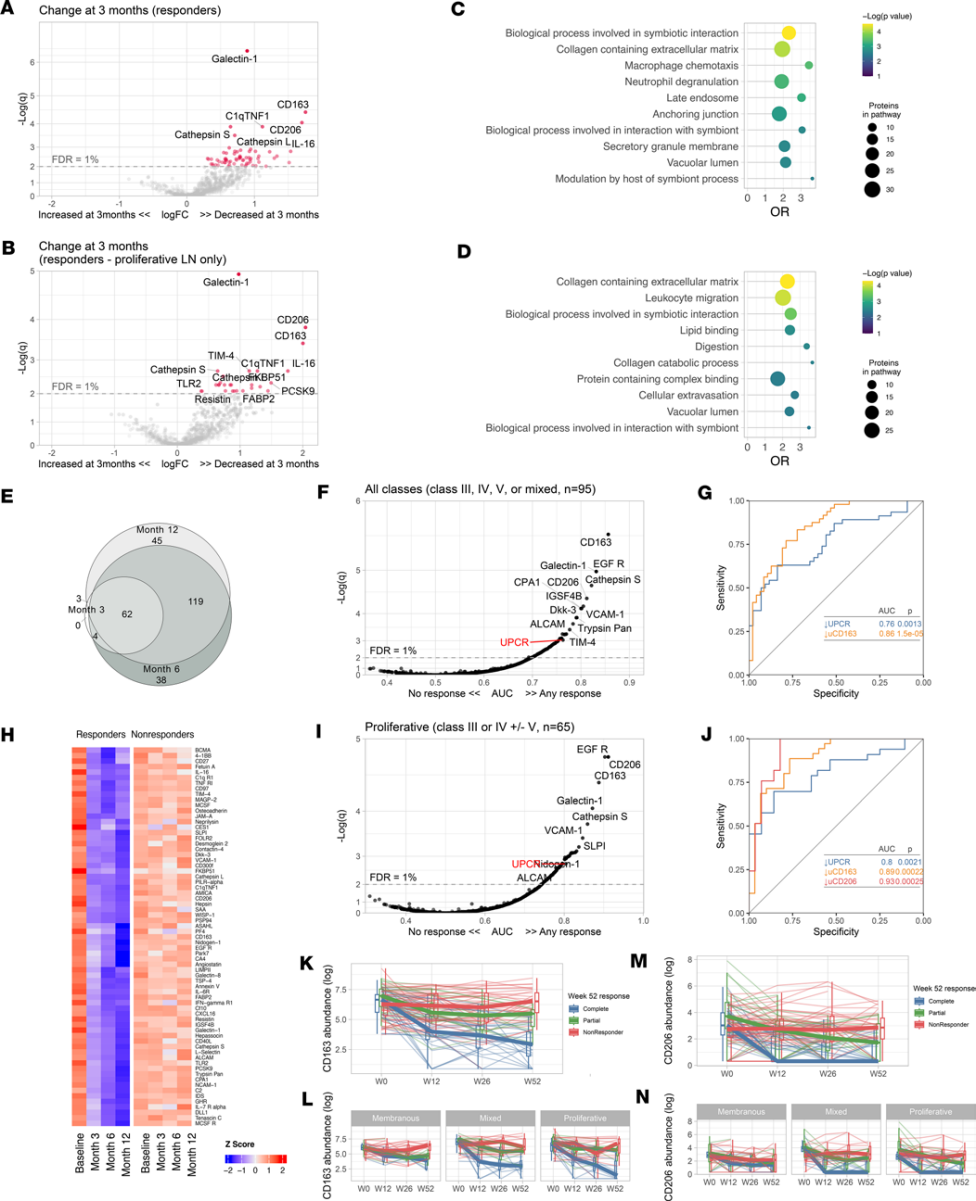
Proteomic changes of treatment response. Volcano plots of the changes of the urinary proteomic profiles of treatment responders at 3 months after kidney biopsy/treatment compared with baseline at time of biopsy in proliferative and membranous combined (**A**) or proliferative only (**B**). (**C** and **D**) Pathway enrichment analysis of the urinary proteins declined in **A** and **B**, respectively. (**E**) Venn diagram summarizing the shared significantly changed proteins at 3, 6, and 12 months after the kidney biopsy. (**F**) Heatmap displaying the urinary abundances of the proteins significantly decreased at 3 months in responders from panel **A** at the 4 time points according to response status. (**G**) Discriminatory power of the change of each urinary protein at 3 months compared with baseline to predict treatment response at month 12 (*n* = 95) (displayed as area under the curve, AUC). The change in UPCR is displayed for reference as the traditionally used biomarker. (**H**) Receiver operating characteristic curves of the decline at 3 months of the UPCR (traditional biomarker) and urinary CD163. **I** and **J** replicate **G** and **H**, but limited to patients with proliferative LN (*n* = 65). (**K**–**N**) Trajectory of the urinary abundance of CD163 (**K** and **L**) and CD206 (**M** and **N**) according to response status in all patients and stratified by ISN class. Thin lines indicate individual trajectories; thick lines indicate the group medians; box plots indicate medians, interquartile range, and range. *q*, adjusted *P* values (Benjamini-Hochberg); OR, odds ratio; FDR, false discovery rate.

To identify pathways that could mediate response to immunosuppression, we studied the changes in the urinary proteome after 3 months of treatment compared with the baseline, according to the response status at 1 year. Responders showed a decline at 3 months in 69 urinary proteins (FDR < 1%) led by galectin-1, CD163, IL-16, and CD206 (Figure 4, A and B). These proteins overlapped with the proteomic signature associated with histological activity (Figure 3A). Accordingly, pathway enrichment analysis after 3 months of treatment showed a decline in pathways related to extracellular matrix and cellular immune response in those who ultimately had a complete or partial response at 1 year (Figure 4, C and D).

The decline of the urine proteins by 3 months persisted at 6 and 12 months. Moreover, an increased number of proteins declined at 6 and 12 months in responders (Figure 4, E and F, and Supplemental Figure 6, B–E). By contrast, there were no changes observed in nonresponders (Figure 4F).

To identify early biomarkers of response, we studied the discriminatory ability of the urinary protein changes at 3 months to predict 1-year response. A total of 111 urinary biomarkers predicted response (FDR < 1%, AUC 0.7–0.86), with most outperforming the improvement in proteinuria (the clinical standard) (Figure 4G). A decline of CD163 at 3 months predicted 1-year response with an AUC of 0.86 (*q* = 2.7 × 10^–6^) compared with an AUC of 0.75 (*q* = 0.01) for proteinuria (Figure 4H). In proliferative LN, urinary biomarkers displayed superior performance with an AUC of 0.91 (*q* = 1.6 × 10^–5^), 0.9 (*q* = 1.6 × 10^–5^), 0.89 (*q* = 3.5 × 10^–5^), and 0.76 (*q* = 0.007) for the decline of CD206, EGFR, CD163, and proteinuria, respectively (Figure 4, I and J). In pure membranous LN, Smad4 and LAMA4 displayed AUCs of 0.88 and 0.75, respectively, with nominal *P* < 0.01 but *q* > 0.7. There were no biomarker changes at 3 months predicting response in pure membranous LN that reached statistical significance after correcting for multiple comparisons.

These findings indicate that effective immunosuppression induces an immunological response in the kidney by 3 months that can be noninvasively monitored in the urine. Because proliferative LN is characterized by the infiltration of intraglomerular myeloid immune cells, a decline in urinary biomarkers of myeloid inflammation in responders suggests a parallel resolution of intraglomerular inflammation. This was specific to proliferative LN as exemplified by the trajectories of CD163 and CD206 (Figure 4, K–N).

## Discussion

The discovery of disease mechanisms, patient subgroups, biomarkers, and novel targets can be simultaneously derived from the analysis of careful phenotypes, longitudinal trajectories, and differential outcomes associated with specific interventions (19). Here, we leveraged urine proteomics to discover 1) LN biology and pathways of LN activity and 2) biomarkers of disease activity and treatment response. We identified that neutrophil/monocyte degranulation, macrophage activation, and extracellular matrix degradation are implicated in LN activity. These processes can be noninvasively quantified and monitored in urine. Reduction in the signatures of these processes at 3 months predicted treatment response. These noninvasive urine biomarkers (such as CD163 and CD206) that parallel intrarenal inflammation outperformed the current clinical standard (proteinuria). Furthermore, this study validated IL-16 as the urinary biomarker most correlated with LN activity (9), supporting its role as a potentially novel therapeutic target and biomarker.

### LN biology and pathways of activity.

Protease activity and extracellular matrix remodeling were shared by pure membranous and proliferative classes, indicating that even the less inflammatory class V LN undergoes kidney remodeling. Proliferative classes were characterized by stronger macrophage and degranulation signatures that correlated with histological activity. Macrophages are the dominant immune cell type in LN (20). CD163 (hemoglobin receptor) and CD206 (mannose receptor) exist in soluble forms as they are shed during inflammation (21). In our analysis, urinary CD163 and CD206 were increased in all classes (but at higher levels in proliferative LN), they correlated with the NIH Activity Index, and their decline best predicted treatment response. Similarly, the intrarenal abundance of CD163^+^ and CD206^+^ macrophages correlated with LN histopathological indices of LN activity (20, 22). These findings 1) validate the association between injury-associated macrophages and LN activity and 2) indicate that the disappearance of these macrophages or their differentiation to a different phenotype anticipates better outcomes.

Several neutrophil/monocyte granule proteins (i.e., PR3 and azurocidin) in the urine were linked to LN activity, implicating degranulation in proliferative LN. Azurophil granules characterize neutrophils and monocytes (23). Neutrophils, especially the subset of low-density granulocytes, have been widely implicated in SLE pathogenesis and LN (24–26). Intraglomerular neutrophils and karyorrhectic debris from apoptotic neutrophils are in fact a feature of proliferative LN and are scored in the NIH Activity Index (18). However, mature neutrophils with classical polylobate nuclei are not a dominant cell type observed in LN kidney biopsies. Rather, immature forms of neutrophils implicated in the pathogenesis of LN (27, 28) do not have polylobate nuclei, suggesting that their presence in LN kidney may not be noted with traditional light microscopy (28). These less mature forms of granulocytes have enhanced ability to degranulate (28), suggesting a potential role in LN. PR3 can in fact lead to extracellular matrix degradation, which, in turn, can lead to fibrosis and irreversible kidney damage (29–31). Our study demonstrates the ability of urine proteomics to explore a wide array of pathological processes, including neutrophil biology, which can be missed in cellular studies involving sample freezing (32). Further studies are needed to define the main cell type responsible for degranulation in LN (neutrophils, monocytes, or other myeloid cells) but also to discover if this urinary signature reflects intrarenal degranulation or spillage of circulating granule proteins.

We have previously discovered that urinary IL-16 is the protein most correlated with the NIH Activity Index (9). Here, we validated this finding, applying an unbiased approach in an independent larger cohort of patients with LN, corroborating the role of IL-16 as a biomarker and in LN pathogenesis. The association of urinary IL-16 with active proliferative LN was also validated in an independent Swedish cohort (33). IL-16 is a proinflammatory chemokine that can activate and recruit CD4^+^ and CD9^+^ cells (34–37). Pro–IL-16 is cleaved into bioactive IL-16 by caspase-3 (36) or PR3 (38), indicating that both cell death and neutrophil/monocyte degranulation could lead to IL-16 activation. Notably, CD9 controls migration and proliferation of parietal epithelial cells in response to podocyte injury (39). CD9 stimulation mediates glomerular crescent formation and glomerular demolition (39), thereby linking IL-16 to a nonimmune mechanism of proliferative LN associated with poor renal survival and mortality in LN (40, 41).

Collectively, the findings from this work indicate that the active phase of proliferative LN is characterized by degranulation, phagocytic/injury-associated macrophage activation, chemokine release, and extracellular matrix degradation. We speculate that IL-16 may play a central role in fueling inflammation by attracting more immune cells such as neutrophils/monocytes and promoting crescent formation. Neutrophil or monocyte degranulation may directly damage the glomerular endothelium (42) and remodel extracellular matrix, promoting chronic kidney disease. It is unclear whether phagocytic and injury-associated macrophages play a regulatory or proinflammatory role in the initial phase of LN activity. Nevertheless, their disappearance or their differentiation to a different phenotype is associated with treatment response, suggesting that they track with the resolution of inflammation. Importantly, these pathogenic processes can be noninvasively monitored in the urine.

Despite fibrous crescents being considered inactive lesions that follow crescentic glomerulonephritis, urine proteomics revealed inflammatory activity associated with fibrous crescents. Thus, the presence of fibrous crescents in kidney biopsies may indicate ongoing potentially treatable inflammation. In fact, crescents are classified as fibrous when composed of < 25% of cells and fibrin, and therefore a small inflammatory infiltrate can be part of fibrous crescents (18). Interstitial inflammation, which is linked to worse clinical outcomes (43), showed a distinct proteomic signature combining both activity and chronicity. This is likely because the current classification system does not separate interstitial inflammation occurring in areas with or without fibrosis. Interstitial fibrosis is in fact frequently infiltrated by immune cells. These results challenge the current interpretation of histological scores. A better understanding of the pathophysiology of processes including fibrous crescents and interstitial inflammation is needed to tailor treatment of these pathways leading to chronic damage.

### Biomarkers.

This work demonstrated the feasibility of urine biomarkers to noninvasively predict clinically meaningful outcomes. Previous unbiased studies focused on the identification of biomarkers to diagnose LN versus no LN (14, 15, 44, 45). However, LN presence and activity were defined by proteinuria, a readily available biomarker. Therefore, the clinical value of the novel biomarkers over proteinuria could not be established. Other studies identified urinary biomarkers of histological activity or evaluated their longitudinal changes with treatment (15, 44, 45), but these were limited to a few selected candidate biomarkers. Here, 1) we described biomarkers to identify LN histological class and activity in lupus patients with proteinuria, the group in which renal biopsies are sought for diagnosis, and 2) we systematically studied the trajectories of 1,200 potential biomarkers in relation to clinical response. These findings have important diagnostic implications.

The current classification and treatment of LN rely on histological features at the time of biopsy. A higher NIH Activity Index usually triggers more aggressive immunosuppression. In contrast, when there is a low NIH Activity Index in the presence of a high NIH Chronicity Index, proteinuria is considered secondary to damage and, therefore, not requiring new or increased immunosuppression. The unbiased catalog of urinary biomarkers of LN (outperforming proteinuria) in this large cohort of proteinuric patients provides the basis for clinically useful biomarkers that would impact clinical decisions.

Prediction of treatment response is key to improve treatment strategies. Although there were no biomarkers at baseline that predicted response, the decline of several urinary biomarkers after 3 months of treatment strongly predicted response at 1 year. These findings underscore the power of individual trajectories to discover disease biology and to identify clinically meaningful patient subsets. Persistent elevation of the NIH Activity Index in a repeat biopsy is associated with LN flares and 44% 10-year kidney survival, compared with 100% in patients with an index of 0, regardless of resolution of proteinuria (46–49). Therefore, characterization of the pathways involved in LN activity is key to the identification of new treatable targets and biomarkers to guide diagnosis and treatment. Frequent kidney biopsies are not a practical means to judge changes in activity and chronicity indices. However, our proteomic analysis offers a feasible strategy of early and frequent monitoring. Patients with higher activity had higher urinary abundance of biomarkers of inflammation (i.e., IL-16), degranulation (i.e., PR3, azurocidin, catalase, MMP8, LAMP1-2), macrophage activation (i.e., CD163, CD206, galectin-1, cathepsins, MIP-1b), and extracellular matrix degradation (i.e., nidogen-1, collagens, proteoglycans). A reduction in biomarkers of these processes predicted future treatment response and outperformed proteinuria. This suggests that the effective inhibition of pathogenic mechanisms by immunosuppression can be noninvasively monitored in real time. These responses are faster than the resolution of proteinuria, which requires repair of the glomerular capillary wall. A biomarker panel to noninvasively assess intrarenal activity may reshape the treatment strategy of LN based on “immunological responses” (and inform clinical trial design). For example, patients with persistent urinary biomarker elevation (indicating activity regardless of improved proteinuria) would receive more potent, different, or prolonged immunosuppression or a change in approach, while those with normalized urinary biomarker levels (indicating immunologically resolved LN) could continue and eventually safely taper potentially toxic medications. These biomarkers may guide treatment selection and clinical trial design. For example, there are currently several treatment options for LN, but the choice of the best initial treatment strategy remains unclear. In patients where urine proteomics showed no reduction in these predictors of treatment response, treatment could be rapidly modified until an immunological response is achieved without waiting for improvement in proteinuria, which does not track with intrarenal inflammation. Conversely, early immunological responses in the urine proteome can reassure that the current treatment is effective. Future clinical trials and longitudinal studies should address how these urinary biomarkers of intrarenal pathology can guide treatment and whether immunological responses predict long-term preservation of kidney function.

Finally, this study validated several known biomarkers of LN. Among others, urinary CD163 (9, 50, 51), monocyte chemoattractant protein-1 (MCP-1) (52), lipocalin-2 (53), and ALCAM (13) were increased in proliferative LN. Of these, only CD163 and MCP-1 correlated with the NIH Activity Index. EGFR (54) negatively correlated with the NIH Chronicity Index and positively with the NIH Activity Index.

### Limitations.

We acknowledge several limitations. First, although the proteomic assay employed here allowed for the specific and highly sensitive detection of 1,200 targets, other processes might be detected using future proteome-wide broader arrays. Second, the AMP study was an observational cohort, and treatment was not homogenous as in a clinical trial. There may be biomarkers at baseline or at 3 months that better predict response to specific treatments that could not be identified. Future studies involving protocolized treatment are needed to identify drug-specific response signatures (55). Third, LN activity was quantified according to the NIH Activity Index, which is more heavily weighted on glomerular than tubulointerstitial pathology. However, it should be acknowledged that tubulointerstitial disease and other histological features are also linked to kidney survival (43).

### Conclusion.

This study showed that urine proteomics is a powerful tool to discover disease processes, nominate treatable targets, and identify noninvasive biomarkers. This data set generated by the AMP is a publicly available resource for future studies. Deep phenotyping of LN by integration of multiomics such as kidney single-cell RNA-Seq, multiplexed histology, digitalized histology, genetics, blood studies, and other modalities (16) in matching samples will help identify novel biomarkers, LN subgroups, and treatment strategies.

## Methods

### Patients and sample collection.

This study enrolled SLE patients with a UPCR > 0.5 who were undergoing clinically indicated renal biopsy. Only patients with a pathology report confirming LN were included in the study. Renal biopsy sections were scored by a renal pathologist at each site according to the ISN/Renal Pathology Society guidelines and the NIH Activity and Chronicity Indices (18). Clinical information, including serologies, were collected at the most recent visit before the biopsy. Response status at week 52 was defined in patients with a baseline UPCR > 1 as follows: complete response (UPCR ≤ 0.5, normal serum creatinine or < 25% increase from baseline if abnormal, and prednisone ≤ 10 mg daily), partial response (UPCR > 0.5 but ≤ 50% of baseline value, identical serum creatinine, but prednisone dose could be up to 15 mg daily), or no response (UPCR > 50% of baseline value, new abnormal elevation of serum creatinine or ≥ 25% from baseline, or prednisone > 15 mg daily). Urine specimens were acquired on the day of the biopsy (before the procedure) or within 3 weeks of the kidney biopsy. Urine samples were immediately centrifuged (4°C, 193*g*, 10 minutes) to remove the cellular component. Serologic features and complement levels were assessed at the clinical visit preceding the biopsy. Proteinuria was measured on or near the day of the biopsy.

### Urine quantibody assay.

An extended version of the Kiloplex Quantibody (RayBiotech) was used to screen urine samples as previously described (8, 9). Concentration of each analyte was normalized by urine creatinine to account for urine dilution. Urine protein abundances are expressed are pg_protein_/mg_creatinine_.

### Statistics.

Differential protein abundance in 2 groups was calculated using a Wilcoxon rank test in univariate analyses. This nonparametric test allowed for robust analysis accounting for the difference in distribution, which is often not normal, across the 1,200 features. We observed similar performance to logistic regression (Supplemental Figure 7). For multivariable analyses, we used linear models or generalized linear models (R lm and glm functions) after log-transforming the protein abundances (models indicated on top of the figures). To account for sparsity and large variation in the dynamic ranges of the proteins, all values for each protein abundance were added to the minimum measured value before log transformation. Correlations and partial correlations (R ppcor package) were calculated on log-transformed protein abundances.

Pathway enrichment analysis was performed with the clusterProfiler or fgsea R packages using the Gene Ontology and Reactome libraries. Genes coding for the measured proteins were used. Analysis was limited to gene sets with at least 5 genes represented in the universe of the 1,200 proteins measured. To account for a limited universe of proteins (not the whole coding genome), self-contained algorithms were applied. Gene set enrichment analysis is inherently self-contained. To define the pathways enriched in a distinct group of proteins (i.e., Figure 2, A–D), a hypergeometric test was used. Terms with > 75% protein overlap were removed: the term with the lowest *P* value was retained. *P* < 0.05 was considered statistically significant. All statistical tests were 2 sided. All analyses were performed in R version 4.1.2.

### Study approval.

Human study protocols were approved by the institutional review boards (IRBs) at each participating site, and written informed consent was obtained from all participants. Patients were enrolled at Johns Hopkins University; New York University; Albert Einstein College of Medicine; University of Rochester Medical Center; Northwell Health; University of California, San Francisco; Medical University of South Carolina; University of California, San Diego; Cedars-Sinai Medical Center; University of Michigan; Texas University, El Paso; and University of California, Los Angeles. For healthy controls, IRB approval was obtained from the University of Cincinnati and Oklahoma Medical Research Foundation. After receipt of informed consent, controls were recruited at the University of Cincinnati. Samples were stored by the Oklahoma Rheumatic Disease Research Cores Center and were matched for sex, race, ethnicity, and age. Participants were screened using a questionnaire and tested negative for the following antibodies: antinuclear, double-stranded DNA, chromatin, ribosomal P, Ro, La, Smith (Sm), SmRNP, RNP, centromere B, Scl-70, and Jo-1. Samples were processed, stored, and shipped using protocols from the AMP in RA/SLE network to align with the patient samples.

### Data availability.

The complete data sets used in this study will be available on the Synapse platform (https://www.synapse.org; https://doi.org/10.7303/syn53124971) at the time of publication. No custom mathematical algorithm deemed central to the conclusions was generated. Analyses can be reproduced using the publicly available versions of the R packages outlined above. Values for all data points found in graphs are in the Supporting Data Values file.

## Author contributions

AF, MP, and J Buyon were responsible for conceptualization. AF, LM, SR, and AA were responsible for methodology. AF was responsible for formal analysis. AF, MP, J Buyon, JH, AR, DSD, JHA, J Barnas, MD, DW, RF, DK, KK, MGA, JMT, DF, RC, HMB, PI, CP, AIC, JH, and DSD were responsible for resources. WA and DG were responsible for project administration. AF, MP, J Buyon, BD, AD, SR, NH, JAJ, and JG were responsible for supervision. AF wrote the manuscript. MP, J Buyon, DAR, MD, JAJ, MGA, HMB, PH, CCB, AD, and BD were responsible for review and editing.

## Supplementary Material

Supplemental data

Supporting data values

## Version 1 01/23/2024

Electronic publication
