# Supplementary material for: Urine proteomic signatures of histological class, activity, chronicity, and treatment response in lupus nephritis
Source: JCI Insight. 2024 Jan 23;9(2):e172569. doi: 10.1172/jci.insight.172569 (PMC10906224; doi:10.1172/jci.insight.172569)
Supplement: Supplemental data [file jciinsight-9-172569-s096.pdf]

Urine proteomic signatures of histological class, activity, chronicity, and treatment response in lupus nephritis.

Fava A, et al.

Supplementary Table

1. Clinical and demographic characteristics.

Supplementary Figures

1. Network analysis of proteomic profiles of LN classes
2. Protein profiles of LN classes.
3. Network analysis of the pathways correlating with histological activity and chronicity.
4. Multivariable analysis of proteomic signatures of histological activity and chronicity.
5. Proteomic signatures of lupus nephritis histological lesions.
6. Additional proteomics signatures associated with response
7. Models' comparison.

Supplementary Acknowledgments

Table S1

	Overall	HD	Lupus nephritis					p
			I/II	III or IV	III or IV +/- V	V	VI	
n	235	10	21	85	55	55	9	
Age (mean (SD))	36.4 (11.8)	38.3 (14.8)	39.4 (11.9)	34.6 (11.3)	36.7 (11.7)	36.6 (11.5)	41.2 (13.8)	0.394
Sex, F/M (%)	202/33 (86.0/14.0)	8/2 (80.0/20.0)	20/1 (95.2/4.8)	76/9 (89.4/10.6)	44/11 (80.0/20.0)	46/9 (83.6/16.4)	8/1 (88.9/11.1)	0.472
Race (%)								0.499
Asian	35 (14.9)	1 (10.0)	5 (23.8)	13 (15.3)	8 (14.5)	6 (10.9)	2 (22.2)	
Black	99 (42.1)	3 (30.0)	11 (52.4)	32 (37.6)	18 (32.7)	30 (54.5)	5 (55.6)	
White	72 (30.6)	6 (60.0)	4 (19.0)	28 (32.9)	20 (36.4)	13 (23.6)	1 (11.1)	
Other	6 (2.6)	0 (0.0)	1 (4.8)	3 (3.5)	1 (1.8)	1 (1.8)	0 (0.0)	
Unknown	23 (9.8)	0 (0.0)	0 (0.0)	9 (10.6)	8 (14.5)	5 (9.1)	1 (11.1)	
First biopsy (%)	82 (34.9)	0 (0.0)	6 (28.6)	41 (48.2)	20 (36.4)	15 (27.3)	0 (0.0)	0.002
Proliferative class (%)								<0.001
III	78 (33.2)			46 (54.1)	32 (58.2)			
IV	60 (25.5)			39 (45.9)	21 (38.2)			
NIH Activity Index (median [IQR])[1]	4 [1, 8]		1 [0, 2]	5 [3, 9]	7 [5, 11.5]	0 [0, 1]	0 [0, 0]	<0.001
NIH Chronicity Index (median [IQR])[1]	3 [1, 5]		3 [2.5, 4]	2 [1, 4]	3 [2, 5.25]	3 [1, 5.75]	9[3]	0.113
UPCR (mean (SD))	2.72 (2.39)		1.30 (1.09)	2.39 (1.74)	3.62 (3.23)	2.90 (2.42)	2.16 (1.13)	0.002
Serum creatinine mg/ml at biopsy (mean (SD))	1.18 (0.82)	0.76 (0.15)	1.00 (0.43)	1.23 (0.91)	1.21 (0.77)	1.04 (0.61)	2.25 (1.37)	0.001
eGFR ml/min at biopsy (mean (SD))	85.9 (35.9)	109.1 (19.1)	88.6 (33.6)	84.0 (36.3)	83.2 (35.3)	94.2 (34.7)	38.4 (16.7)	<0.001
Low C3 (%)	130 (60.2)		5 (31.2)	65 (79.3)	35 (64.8)	24 (43.6)	1 (11.1)	<0.001
Low C4 (%)	107 (49.5)		7 (43.8)	55 (67.1)	28 (51.9)	17 (30.9)	0 (0.0)	<0.001
Response status[2] (%)								0.28
Complete	34 (14.5)			16 (18.8)	13 (23.6)	5 (9.1)		
Partial	29 (12.3)			11 (12.9)	9 (16.4)	9 16.4)		
No	64 (27.2)			21 (24.7)	20 (36.4)	23 41.8)		

Supplementary Table 1. Clinical and demographic characteristics.

[1] Activity and Chronicity Indices were available for 154 patients.
[2] Response status was defined only for patients with baseline UPCR >1.
[3] Chronicity Index available for only 1 patient.
UPCR: urine protein-to-creatinine ratio.

Figure S1

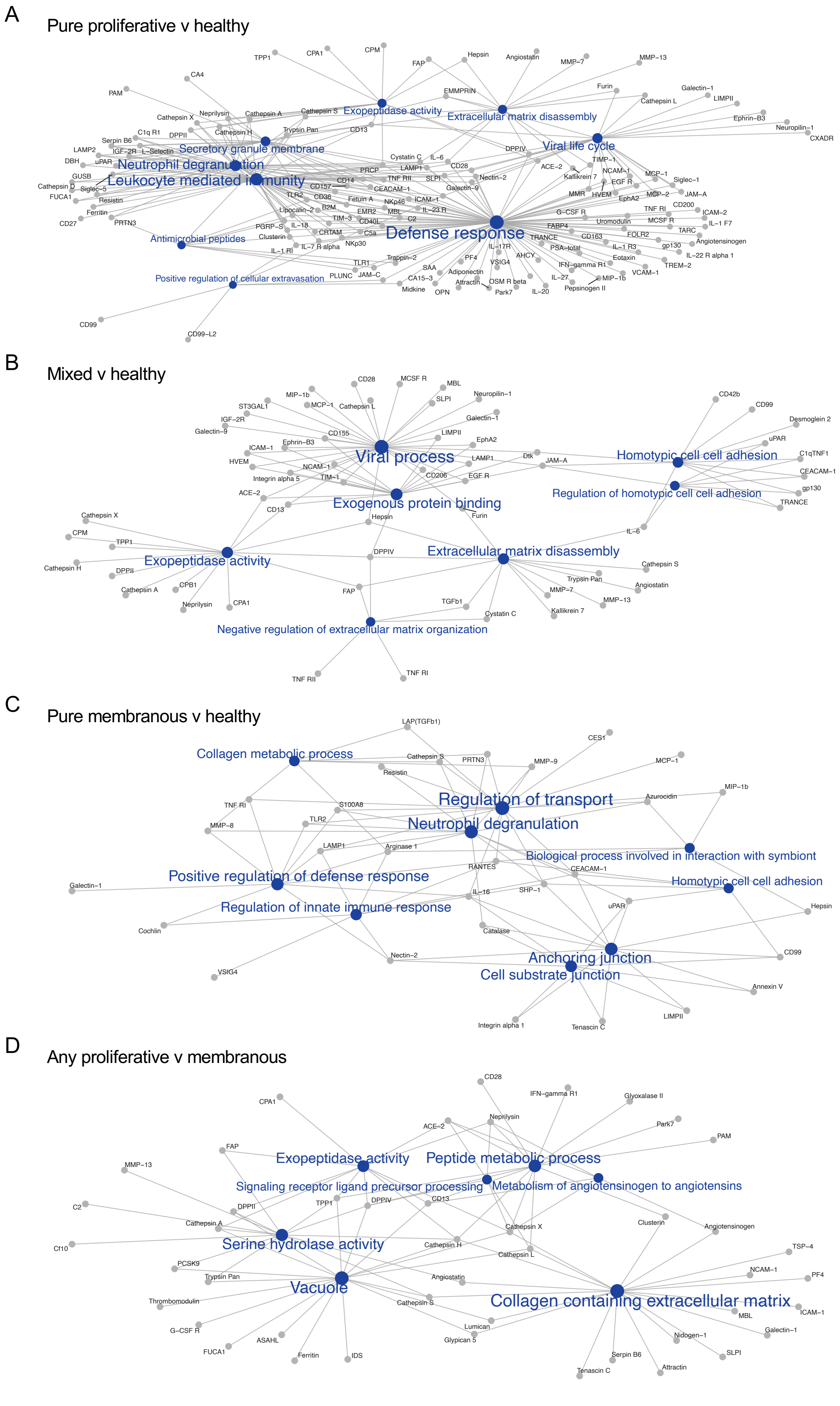

Figure S1. Network analysis of proteomic profiles of LN classes. Network analysis of the top 10 (or all with FDR <5%) enriched pathways comparing the urine proteomic profiles of LN classes with healthy donors or between classes.

Figure S2

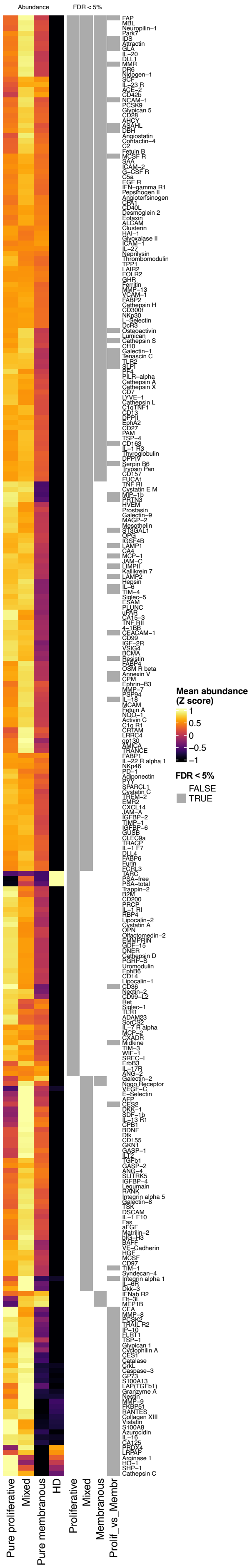

Figure S2. Proteomic profiles of LN classes. Heatmap displaying the urinary abundance (row normalized, Z score) of the proteins significantly enriched (FDR < 5%) in each class (fig. 2A-C) or comparing proliferative with membranous LN (fig. 2I). A gray square in the right panel indicate if the protein was significantly enriched (FDR < 5%) in the urine of the class indicated compared to healthy controls (first 3 columns) or comparing proliferative with membranous LN (last column).

Figure S3. Network analysis of the pathways correlating with histological activity and chronicity

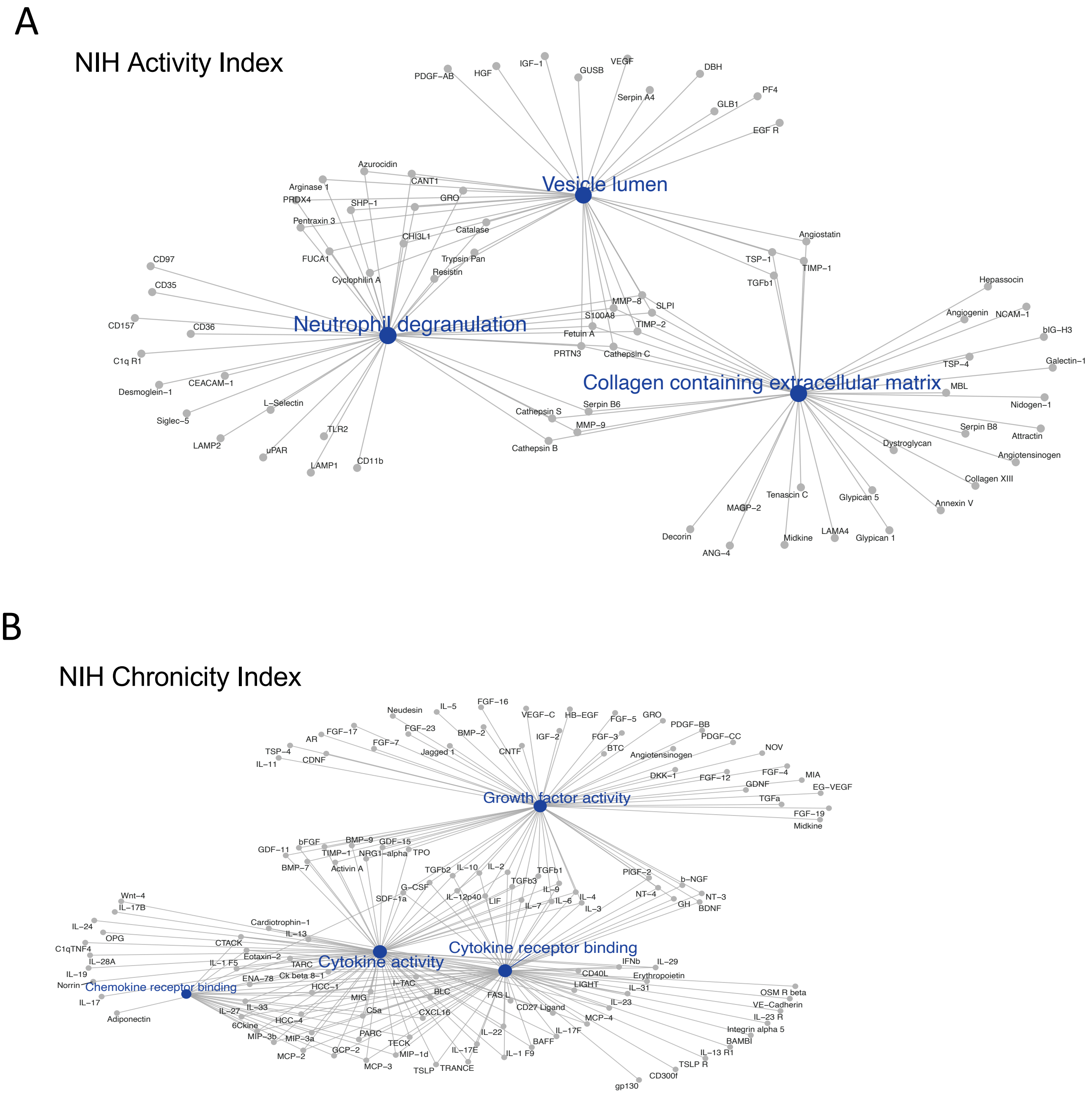

Figure S3. Network analysis of the pathways correlating with histological activity (A) and chronicity (B).

Figure S4. Multivariable analysis of proteomic signatures of histological activity and chronicity.

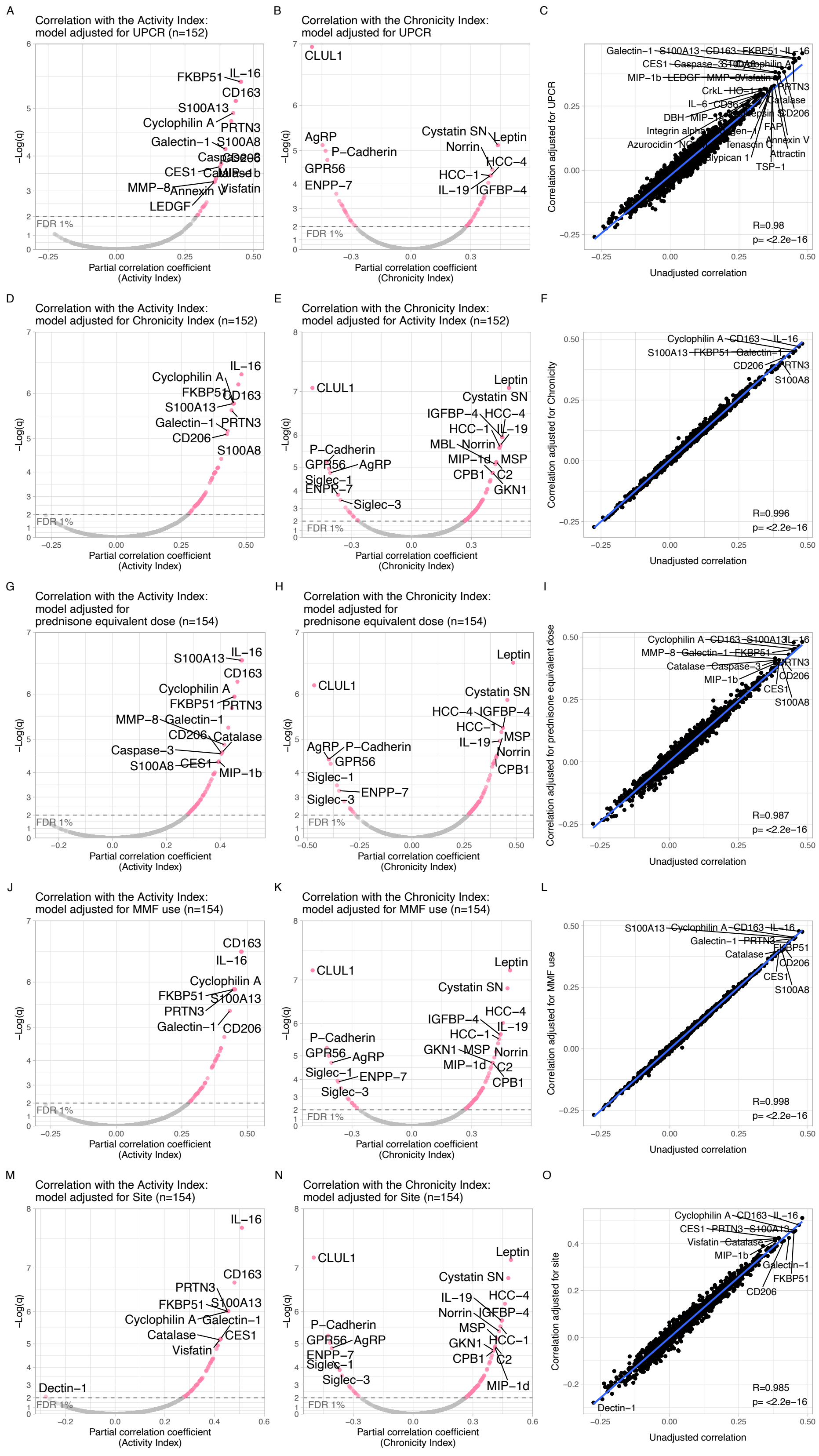

Figure S4. Multivariable analysis of proteomic signatures of histological activity and chronicity. Volcano plots displaying Pearson's partial correlation of the proteins urinary abundances and the NIH Activity and Chronicity indices after adjusting in a multivariable linear model for proteinuria (UPCR) (A-C), the NIH Chronicity or Activity Index (D-F), prednisone equivalent dose (G-I), mycophenolate (MMF) use (J-L), and site (M-O). *FDR=false discovery rate; q=Benjamini-Hochberg adjusted p value.*

Figure S5. Proteomic features of lupus nephritis histological lesions

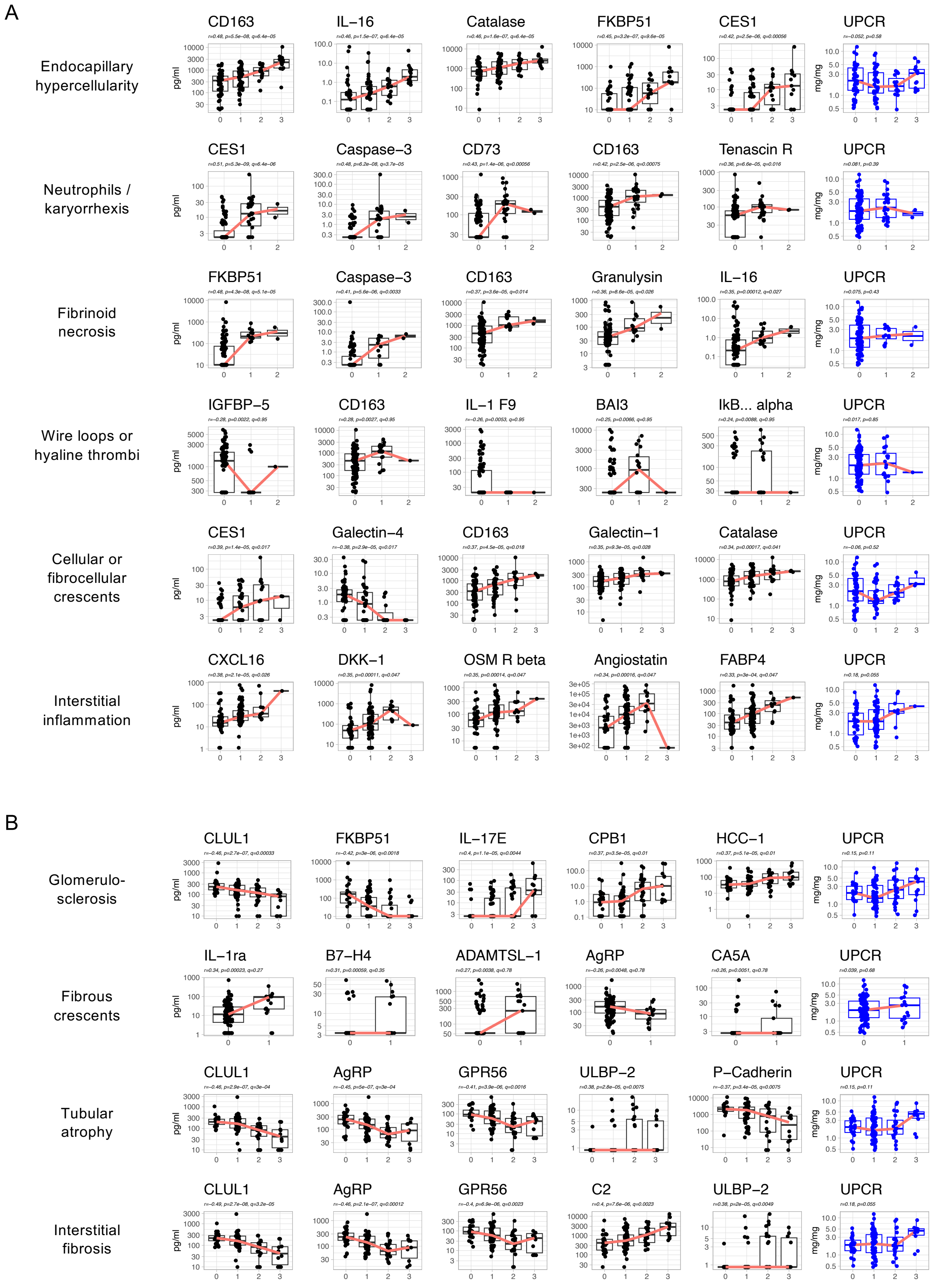

Figure S5. Proteomic signatures of histological activity and chronicity lesions. The 5 most correlated proteins with each histological lesion in the NIH Activity and Chronicity Indices summarized in figure 3F-G are displayed in A and B as compared to UPCR. Spearman's correlation coefficients, p , and adjusted p values (q) are shown)

Figure S6. Additional proteomic signatures associated with response.

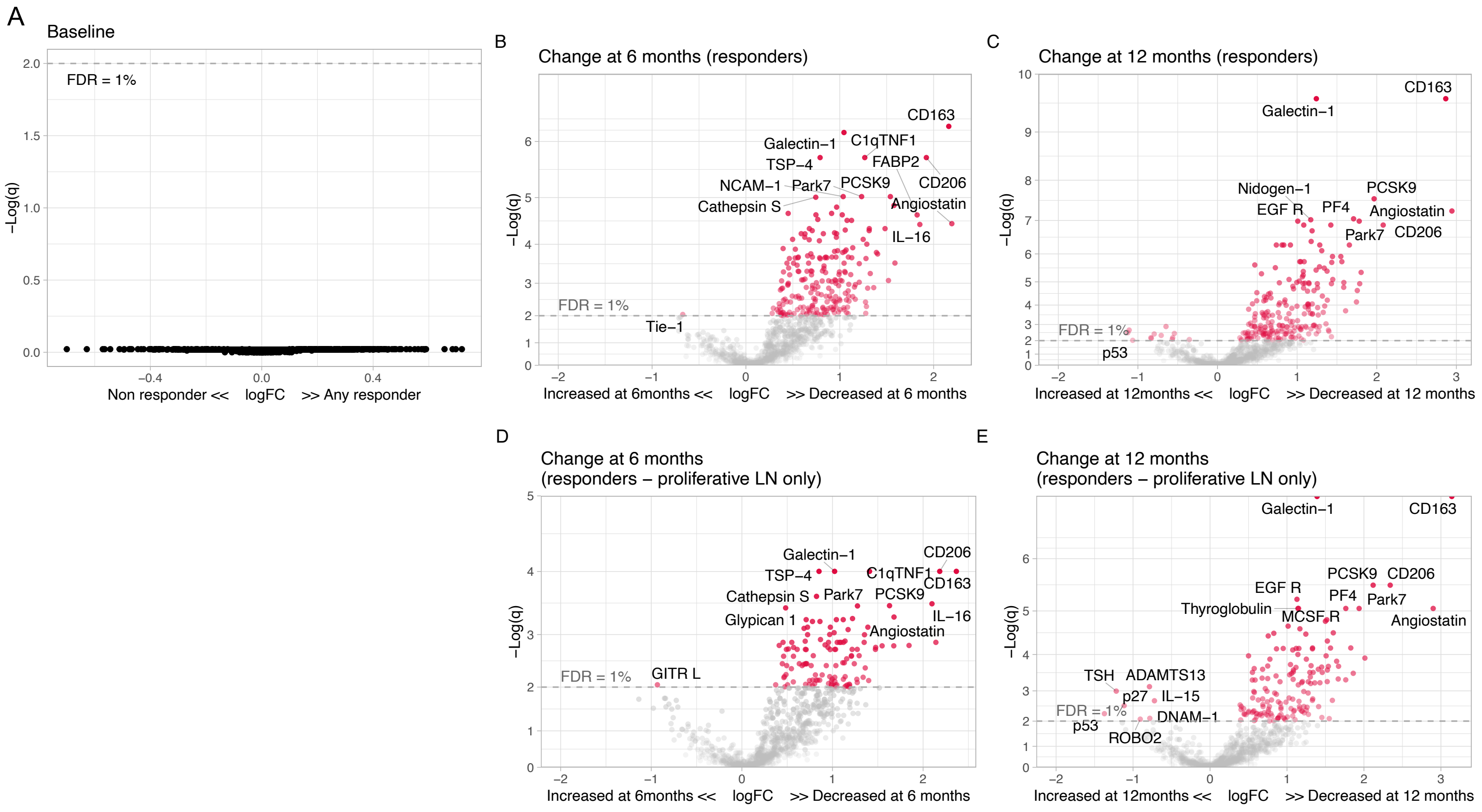

Figure S6. Additional proteomic signatures associated with response. (A) Volcano plots displaying that no urinary protein abundances at baseline were different in 12-month responders compared to non responders. Volcano plots of the changes of the urinary proteomic profiles of treatment responders at 6 (B) and 12 (C) months after kidney biopsy/treatment compared to baseline at time of biopsy in proliferative and membranous combined. D and E replicate panels B and C, but limited to proliferative LN. *q values = adjusted p values (Benjamini-Hochberg). FC = fold change. FDR = false discovery rate.*

Supplementary Fig 7. Models' comparison.

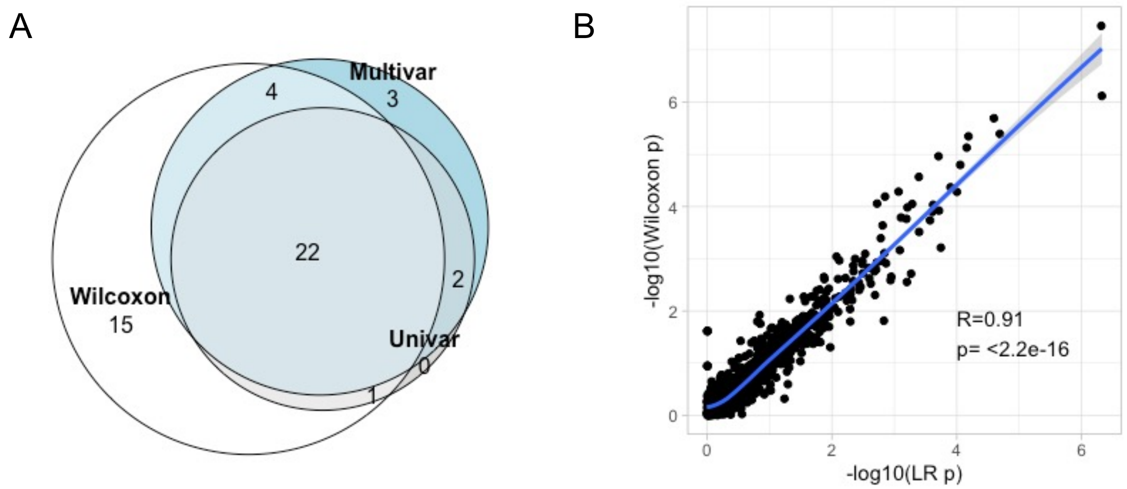

Supplementary Fig 7. Models' comparison. (A) Overlap of differential expressed urinary proteins between any proliferative and pure membranous LN using 3 models: Wilcoxon, univariate logistic regression (class ~ protein_abundance), and multivariate logistic regression (class ~ protein_abundance + UPCR). An FDR of <5% was considered statistically significant. (B) Correlation of p values for the association of each of the urinary protein abundance with the outcome variable (any proliferative or pure membranous LN) using the rank-based Wilcoxon test or logistic regression (LR). Blue line displaying the loess curve. Pearson’s correlation coefficient and p values are displayed.

Supplemental Acknowledgments

Fullname	Institution
**A. Helena Jonsson MD PhD**	Brigham and Women Hospital
**Alessandra Nerviani**	Queen Mary University of London
**Ami Ben-Artzi MD**	Cedars-Sinai Medical Center
**Amit Lakhanpal MD, PhD**	Hospital for Special Surgery, Weill Cornell Medical College
**Andrew Filer MD PhD**	University of Birmingham
**Andrew McDavid PhD**	University of Rochester Medical Center
**Anjali Thakrar**	Northwestern University
**Anvita Singaraju**	Hospital for Special Surgery, Weill Cornell Medical College
**Aparna Nathan**	Harvard University
**Arnold Ceponis**	University of California, San Diego
**Arthur M. Mandolin II MD PhD**	Northwestern University
**Beatrice Goilav**	Einstein Medical College
**Bill Robinson MD PhD**	Stanford University
**Brendan Boyce MD**	University of Rochester Medical Center
**Carla M. Cuda PhD**	Northwestern University
**Christopher Ritchlin MD**	University of Rochester Medical Center
**Costantino Pitzalis MD PhD**	Queen Mary University of London
**Daimon Simmons MD PhD**	Harvard University
**Darren Tabechian MD**	University of Rochester Medical Center
**David A. Hildeman PhD**	Cincinnati Children's Hospital Medical Center
**David L. Boyle**	University of California, San Diego
**David Lieb PhD**	Broad Institute of MIT and Harvard
**Diane Horowitz MD**	Feinstein Institutes for Medical Research
**E. Steve Woodle MD**	Cincinnati Children's Hospital Medical Center
**Edward DiCarlo MD**	Hospital for Special Surgery, Weill Cornell Medical College
**Ellen M Gravallese MD**	Brigham and Women Hospital
**Fan Zhang PhD**	University of Colorado Anschutz
**Felice Rivellese Md PhD**	Queen Mary University of London
**Gary S. Firestein**	University of California, San Diego

**Gerald Watts**	Harvard University
**Gregory Keras**	Harvard University
**Harris Perlman PhD**	Northwestern University
**Heather Sherman**	Hospital for Special Surgery, Weill Cornell Medical College
**Hemant Suryawanshi**	The Rickefeller University
**Holden Maecker PhD**	Stanford University
**Ilfita Sahbudin MD PhD**	University of Birmingham
**Ilya Korsunsky PhD**	Harvard University
**Javier Rangel-Moreno PhD**	University of Rochester Medical Center
**Jen Albrecht**	University of Rochester Medical Center
**Jennifer Goff MPH**	Brigham and Women Hospital
**Jennifer Grossman MD**	University of California, Los Angeles
**Jennifer Seifert, MPH**	University of Colorado Anschutz
**Joan M. Bathon MD**	Columbia University
**Joseph Mears**	Harvard University
**Joshua Keegan**	Harvard University
**Kaitlyn Howard**	Harvard University
**Karen Salomon-Escoto MD**	University of Massachusett
**Karim Raza MD PhD**	University of Birmingham
**Kathryn Weinand**	Harvard University
**Kazuyoshi Ishigaki MD PhD**	Harvard University
**Kevin Deane MD PhD**	University of Colorado Anschutz
**Kevin Wei MD PhD**	Brigham and Women Hospital
**Larry W. Moreland MD**	University of Colorado Anschutz
**Laura Donlin PhD**	Hospital for Special Surgery, Weill Cornell Medical College
**Laura Hughes MD**	University of Alabama at Birmingham
**Lindsy Forbess MD MSc**	Cedars-Sinai Medical Center
**Lorien Shakib**	Hospital for Special Surgery, Weill Cornell Medical College
**Mandy McGeachy PhD**	University of Pittsburgh
**Manjunath Kustagi**	The Rickefeller University
**Matthias Kretzler MD**	University of Michigan
**Maureen A. McMahon MD**	University of California, Los Angeles

**Melanie Smith MD, PhD**	Hospital for Special Surgery, Weill Cornell Medical College
**Michael Peters**	Broad Institute of MIT and Harvard
**Michele Bombardieri**	Queen Mary University of London
**Myles Lewis**	Queen Mary University of London
**Nghia Millard**	Harvard University
**Nida Meednu PhD**	University of Rochester Medical Center
**P.J. Utz MD**	Stanford University
**Patrick J. Dunn PhD**	Immport
**Pavel Morozov**	The Rickefeller University
**Peter K. Gregersen MD**	Feinstein Institutes for Medical Research
**Qian Xiao**	Harvard University
**Raji Menon**	University of Michigan
**Raymond Hsu**	University of California, San Francisco
**Rong Mao PhD**	Stanford University
**S. Louis Bridges Jr MD PhD**	Hospital for Special Surgery, Weill Cornell Medical College
**Salina Dominguez PhD**	Northwestern University
**Saori Sakaue**	Harvard University
**Susan Goodman MD**	Hospital for Special Surgery, Weill Cornell Medical College
**Susan Macwana**	Oklahoma Medical Research Foundation
**Thomas Eisenhaure MD PhD**	Broad Institute of MIT and Harvard
**Thomas Tuschl PhD**	The Rickefeller University
**Tony Jones**	Broad Institute of MIT and Harvard
**V. Michael Holers MD**	University of Colorado Anschutz
**Vivian Bykerk MD**	Hospital for Special Surgery, Weill Cornell Medical College
**Yuhong Li**	Brigham and Women Hospital
